# Extended‐spectrum β‐lactamase‐producing and carbapenem‐resistant Enterobacterales bloodstream infection after solid organ transplantation: Recent trends in epidemiology and therapeutic approaches

**DOI:** 10.1111/tid.13881

**Published:** 2022-06-28

**Authors:** Elena Pérez‐Nadales, Mario Fernández‐Ruiz, Belén Gutiérrez‐Gutiérrez, Álvaro Pascual, Jesús Rodríguez‐Baño, Luis Martínez‐Martínez, José María Aguado, Julian Torre‐Cisneros

**Affiliations:** ^1^ Spanish Network for Research in Infectious Diseases (REIPI), Centro de Investigación Biomédica en Red de Enfermedades Infecciosas (CIBERINFEC) Instituto de Salud Carlos III Madrid Spain; ^2^ Infectious Diseases (GC‐03) and Clinical and Molecular Microbiology (GC‐24) Groups, Maimonides Biomedical Research Institute of Cordoba (IMIBIC), Reina Sofía University Hospital, University of Cordoba Cordoba Spain; ^3^ Clinical Units of Infectious Diseases and Microbiology, Reina Sofía University Hospital, University of Cordoba Cordoba Spain; ^4^ Department of Agricultural Chemistry, Edaphology and Microbiology, and Department of Medicine University of Cordoba Cordoba Spain; ^5^ Department of Medicine Unit of Infectious Diseases, “12 de Octubre” University Hospital, Instituto de Investigación Hospital “12 de Octubre” (imas12), Universidad Complutense Madrid Spain; ^6^ Departments of Microbiology and Medicine Clinical Unit of Infectious Diseases and Microbiology, Virgen Macarena University Hospital, Institute of Biomedicine of Seville (IBIS), CSIC, University of Seville Seville Spain

**Keywords:** antibiotics, carbapenem‐resistant Enterobacterales, extended‐spectrum β‐lactamase‐producing Enterobacterales, INCREMENT‐SOT Project, solid organ transplantation, treatment

## Abstract

**Background:**

Infections caused by multidrug‐resistant gram‐negative bacilli (MDR GNB), in particular extended‐spectrum β‐lactamase‐producing (ESBL‐E) and carbapenem‐resistant Enterobacterales (CRE), pose a major threat in solid organ transplantation (SOT). Outcome prediction and therapy are challenging due to the scarcity of randomized clinical trials (RCTs) or well‐designed observational studies focused on this population.

**Methods:**

Narrative review with a focus on the contributions provided by the ongoing multinational INCREMENT‐SOT consortium (ClinicalTrials identifier NCT02852902) in the fields of epidemiology and clinical management.

**Results:**

The Spanish Society of Transplantation (SET), the Group for Study of Infection in Transplantation of the Spanish Society of Infectious Diseases and Clinical Microbiology (GESITRA‐SEIMC), and the Spanish Network for Research in Infectious Diseases (REIPI) recently published their recommendations for the management of MDR GNB infections in SOT recipients. We revisit the SET/GESITRA‐SEIMC/REIPI document taking into consideration new evidence that emerged on the molecular epidemiology, prognostic stratification, and treatment of post‐transplant ESBL‐E and CRE infections. Results derived from the INCREMENT‐SOT consortium may support the therapeutic approach to post‐transplant bloodstream infection (BSI). The initiatives devoted to sparing the use of carbapenems in low‐risk ESBL‐E BSI or to repurposing existing non‐β‐lactam antibiotics for CRE in both non‐transplant and transplant patients are reviewed, as well as the eventual positioning in the specific SOT setting of recently approved antibiotics.

**Conclusion:**

Due to the clinical complexity and relative rarity of ESBL‐E and CRE infections in SOT recipients, multinational cooperative efforts such as the INCREMENT‐SOT Project should be encouraged. In addition, RCTs focused on post‐transplant serious infection remain urgently needed.

## 1 INTRODUCTION

Multidrug‐resistant gram‐negative bacilli (MDR GNB), including extended‐spectrum β‐lactamase‐producing (ESBL‐E) and carbapenem‐resistant Enterobacterales (CRE), are prevalent infectious agents following solid organ transplantation (SOT).^1^, ^2^, ^3^ Infections by MDR GNB are associated with increased risk for recurrent infection, allograft dysfunction, and mortality as compared to infections due to non‐resistant pathogens.^3^, ^4^ In addition, these infections are frequently treated with old or novel antimicrobials for which scarce data on safety and efficacy are available.^3^ Such circumstances highlight the need to improve the clinical management of ESBL‐E and CRE infections among SOT recipients.

In 2018, the Spanish Society of Transplantation (SET), the Group for Study of Infection in Transplantation of the Spanish Society of Infectious Diseases and Clinical Microbiology (GESITRA‐SEIMC), and the Spanish Network for Research in Infectious Diseases (REIPI) published their recommendations on the management of MDR GNB infections in SOT recipients.^3^ This consensus document covers a wide range of relevant aspects of clinical management, from microbiological diagnosis to therapeutic challenges.^3^ Since the release of the SET/GESITRA‐SEIMC/REIPI document, additional works have reviewed management principles of MDR GNB in the setting of SOT,^5^, ^6^ including the recently published guidelines from the Infectious Diseases Community of Practice of the American Society of Transplantation.^5^ In addition, the European Society of Clinical Microbiology and Infectious Diseases (ESCMID) has also issued its guidelines, although not restricted to transplant patients.^7^

**FIGURE 1:**
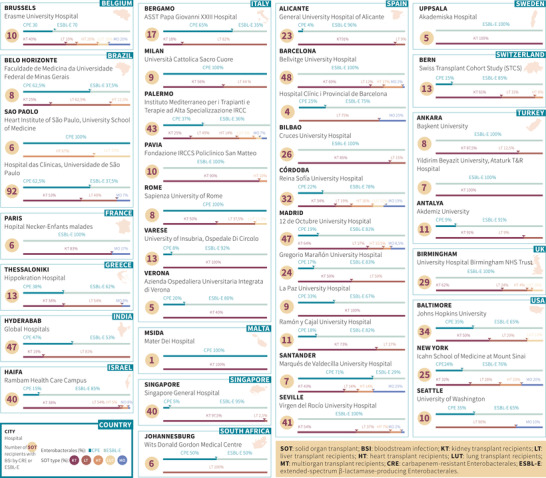
The INCREMENT‐solid organ transplantation (SOT) Project is a multinational consortium including 38 SOT centers. This Project generated a large international retrospective cohort of 788 consecutive recipients with bloodstream infection (BSI) due to extended‐spectrum β‐lactamase‐producing (ESBL‐E) and carbapenem‐resistant Enterobacterales (CRE) for the period 2004 to 2016 (ClinicalTrials identifier NCT02852902)

The INCREMENT‐SOT Project is a large international retrospective cohort that includes nearly 800 consecutive SOT recipients diagnosed with bloodstream infection (BSI) due to ESBL‐E and CRE between 2004 and 2016 (ClinicalTrials identifier NCT02852902).^8^, ^9^, ^10^, ^11^ The INCREMENT‐SOT consortium is currently integrated by 38 tertiary centers in 16 countries (Figure 1), and this cohort study has been recently extended to cover the period 2016–2021. In addition, 14 centers in seven countries from the consortium (seven hospitals in Spain, two in Italy, one in Belgium, one in the UK, one in Turkey, one in Malta, and one in the USA) have participated in the generation of an international microbial collection of 174 ESBL‐E and CRE isolates, representing 20% of the post‐transplant BSI episodes recorded in the clinical database. The study is mainly aimed at providing high‐level observational data on the molecular epidemiology and therapeutic management of ESBL‐E and CRE BSI in the SOT setting. Other aspects, such as pre‐transplant screening, prophylaxis, or decolonization strategies, are outside the intended scope of the INCREMENT‐SOT Project.

In the present work, we review the main contributions made by the INCREMENT‐SOT consortium and summarize additional recent evidence concerning epidemiological trends, risk factors, use of predictive scores to guide clinical management, and new therapeutic alternatives among SOT recipients with ESBL‐E and CRE infection. To this end, we conducted a set of computer‐based PubMed/MEDLINE (National Library of Medicine, Bethesda, MD) searches using the MeSH terms “SOT” (and specific transplant types) AND “extended‐spectrum β‐lactamase” or “carbapenemase‐producing” (or “carbapenem‐resistant”) AND *“Enterobacteriaceae*” (or “Enterobacterales”) AND “infection” or “bacteremia” (and specific syndromes) to identify literature pertaining to the topic published until January 2022. In addition, separate searches were performed for each of the antibiotic agents reviewed. Only clinical studies covering molecular epidemiology and therapeutic aspects were considered. The bibliographies of the selected articles were scrutinized for additional relevant references.

## 2 ESBL‐E INFECTIONS AFTER SOT: EPIDEMIOLOGY, MOLECULAR CHARACTERIZATION, AND CLINICAL FEATURES

The rates of ESBL‐E infection in SOT recipients show geographical variations and have increased in recent years. Approximately 40% of infectious episodes caused by Enterobacterales after SOT are due to ESBL‐E, primarily *Escherichia coli* and *Klebsiella pneumoniae*.^12^, ^13^, ^14^, ^15^, ^16^ A systematic review involving 1089 recipients reported that as much as 18% of them were colonized with ESBL‐E (95% confidence interval [CI]: 5%–36%). The prevalence of ESBL‐E colonization for pre‐transplant candidates was estimated at 7% (95% CI: 5%–9%). Stratifying by transplant type, the ESBL‐E colonization rate was 17% for liver transplant (LT) recipients and 24% for kidney transplant (KT) recipients. According to the continent, no significant differences in the prevalence were identified between studies performed in Europe and North America (15% and 31.4%, respectively).^4^

Molecular characterization of the INCREMENT‐SOT strain collection corresponding to 174 recipients with BSI classified 75% of isolates as ESBL‐E and 25% as CRE (10% of which harbored both ESBL and carbapenemase genes). *E. coli* (53.0%) *and K. pneumoniae* (40.9%) were the most frequent ESBL‐E, whereas *K. pneumoniae* (83.7%) accounted for most CRE isolates. *bla*_CTX‐M_ β‐lactamase genes were the most frequent among ESBL producers, while *bla*_KPC_ and *bla*_OXA‐48_ were the more prevalent carbapenemase genes.^17^ Our data are consistent with those obtained from a prospective cohort study conducted between 2014 and 2018 in seven Spanish centers (ENTHERE study), which reported 78.0% of ESBL‐E and 21% of CRE among 531 isolates from LT and KT recipients.^16^ In this study, *E. coli* producing cefotaximase‐Munich (CTX‐M)‐1 group and *K. pneumoniae* harboring *bla*_OXA‐48_ (either alone or in combination with *bla*_CTX‐M‐1_) were the most prevalent Enterobacterales causing colonization.^16^ A further study analyzing the etiology of post‐transplant BSI in a Spanish center detected an increasing rate of ESBL‐producing strains, mainly *K. pneumoniae*, from 7% in the period 2007–2008 to 34% in 2015–2016.^2^ A nationwide study carried out in Switzerland reported that the proportion of ESBL‐E infections in SOT recipients rose in recent years to 11.4%, although the proportion of ESBL produces among *E. coli* isolates remained stable over time (in contrast to the increasing trend observed for non‐*E. coli* strains).^18^

Risk factors for developing post‐transplant ESBL‐E infection include previous antibiotic exposure, pre‐transplant colonization, perioperative prophylaxis, prolonged tracheal intubation, long‐term hospitalization, urinary tract obstruction and instrumentation, kidney‐pancreas transplantation, post‐transplant renal replacement therapy (RRT), and recurrent urinary tract infection (UTI), as reviewed elsewhere.^3^, ^19^ A recent multicenter case‐control study in the USA comprising 988 ESBL‐E BSI episodes identified various risk factors of relevance, including the isolation of ESBL‐E in a prior culture, a corticosteroid‐containing immunosuppression regimen, acute rejection treated with corticosteroids, and previous exposure to third‐generation cephalosporins, echinocandins and trimethoprim‐sulfamethoxazole.^20^

The incidence of ESBL‐E infection among LT recipients has been reported to reach 5.5%–13%.^13^, ^21^, ^22^ The median time from transplantation to infection was reported to be 15 days (range: 3–105 days) in a unicentric retrospective cohort in France that included 710 patients between 2001 and 2010.^22^ Since the urinary tract is one of the most frequent sources of infection, KT recipients are exposed to a higher incidence. According to a meta‐analysis, the proportion of KT recipients that developed a UTI due to ESBL‐E was 2% in North America, 5% in Europe, 17% in South America, and 33% in Asia. These patients face an almost three‐times increased risk of recurrence compared to those infected with non‐ESBL‐producing strains.^23^ In a 3‐year, monocentric retrospective study in France on 659 KT recipients, the median time from transplantation to the occurrence of UTI due to ESBL‐E was 48 days (range: 17–253).^24^

The most common sources of post‐transplant BSI by ESBL‐E are intravascular catheters and complicated UTI (cUTI).^25^ In a large single‐center retrospective study in Brazil, BSI was more frequent among LT recipients (7% of 238 patients) than KT recipients (3% of 759 patients; *p*‐value = .005).^26^ In this study, the 30‐day mortality rate among 54 recipients that developed ESBL‐E BSI was 26%. However, the proportion of cases of bacteremia secondary to cUTI was significantly higher among KT than LT recipients (60% vs. 11%, *p*‐value = .008), and the 30‐day mortality rate in these patients was 6.7% only.^26^ In the INCREMENT‐SOT cohort, we reported a 30‐day all‐cause mortality rate of 2.9% in the subcohort of 306 KT recipients with non‐severe monomicrobial ESBL‐E BSI secondary to UTI between 2004 and 2016.^11^ Consistent with this more favorable outcome, in a second analysis comparing the efficacy of meropenem versus ertapenem in KT recipients with non‐severe bacteremic urinary tract infections due to ESBL‐E, only one meropenem‐treated patient died within the first 30 days from BSI onset.^9^

Improved outcome prediction in a complex clinical setting such as MDR GNB infection after SOT is critical in order to inform the decision‐making process. Wang et al. recently developed a clinical prediction tool for post‐transplant ESBL‐E BSI on the basis of a multicenter cohort with 897 patients. They generated a predictive model consisting of 10 variables, which fell into four clinical categories: prior colonization or infection with Enterobacterales, recent antimicrobial exposure, the severity of underlying illness, and type of immunosuppression. The authors recommend that any patient scoring 2 or more points should be deemed at high risk of ESBL‐E BSI and considered for empiric carbapenem therapy while awaiting the results of susceptibility testing.^27^

The multinational INCREMENT project preceded INCREMENT‐SOT and was focused on infections due to ESBL‐E and CRE in the general population (ClinicalTrials.gov Identifier NCT01764490). As part of this study, an easy‐to‐collect predictive scoring model for 30‐day all‐cause mortality in ESBL‐E BSI was developed, which has been proven to accurately stratify patients with this condition.^28^ Key risk factors included in this score were: age >50 years (3 points), infection due to *Klebsiella* spp. rather than other species (2 points), a source of infection other than urinary tract infections (3 points), and ultimately or rapidly fatal disease according to the McCabe classification (i.e., death is expected to occur in <5 years as a consequence of the underlying condition, 4 points), a condition of acute severity as measured by a Pitt score >3 (3 points), presentation of BSI with severe sepsis or septic shock (4 points), and inappropriateness of early targeted therapy (2 points). Mortality rates for INCREMENT‐ESBL scores <11 and >11 points were near 5% and 40%, respectively.^28^ The predictive accuracy of this score for risk stratification in SOT recipients remains to be investigated.

## 3 CRE INFECTIONS IN THE SOT POPULATION

The emergence of CRE constitutes a major threat for SOT recipients since infections caused by these difficult‐to‐treat pathogens lead to significant morbidity and mortality.^3^ Mortality rates reported for CRE infections following SOT vary from 20% to 80% and are significantly higher in the case of BSI as compared to non‐bacteremic syndromes.^29^, ^30^, ^31^, ^32^, ^33^ Post‐transplant CRE infections tend to occur within the first two months after the procedure, and the infection site frequently correlates with the transplant type.^3^, ^34^ Thus, LT recipients commonly develop the surgical site (SSI) and intraabdominal infection (IAI), which are often associated with BSI. On the other hand, UTI and SSI are more common among KT recipients, with a high relapse rate. Finally, lung transplant (LuT) recipients are typically at risk of developing pneumonia.^3^, ^5^

The molecular epidemiology of CRE in the SOT setting has been characterized in several recent studies. As part of the INCREMENT‐SOT project, we performed a comparative genomic analysis of 35 carbapenemase‐producing *K. pneumoniae* (CP‐KP) strains from BSI episodes in nine centers (five in Spain, one in Italy, one in Belgium, one in Malta, and one in the USA). *bla*_KPC_ was detected in 68.6% of isolates, in association with sequence type 512 (ST512) (40%, Italy and Spain), ST258 (20%, Italy and USA), ST307 (6%, Italy), and ST101 (3%, Italy). In addition, *bla*_OXA‐48_ was detected in 28.6% of CP‐KP isolates, in association with ST15 (11%, Spain and Belgium), ST11 (9%, Spain), ST392 (3%, Spain), ST405 (3%, Spain), and ST496 (3%, Malta). Finally, *bla*_VIM_ was found in a single *K. pneumoniae* isolate of ST1 (3%, Spain).^17^ Clancy et al. described a series of 17 recipients with CRE BSI from a single US center, with 16 of them infected with the sequence‐type 258 (ST258) harboring the *bla*_KPC‐2_ gene and the remaining patient with a KPC‐3‐producing ST37 strain.^35^ In a prospective cohort study in another US center, Macesic et al. performed a comparative genomic analysis of 95 CRE isolates (57 *K. pneumoniae*, 20 *E. coli*, 15 *Enterobacter cloacae* complex, two *Citrobacter freundii*, and one *K. oxytoca*) from LT recipients with either colonization (80 isolates) or infection (15 isolates). They identified 26 CRE clades, with *K. pneumoniae* ST258 being the most common (39%), followed by *E. cloacae* complex ST252 (8%), *K. pneumoniae* ST17 (7%), and *K. pneumoniae* ST307 (5%). The *bla*_KPC_ gene was detected in 66% of isolates.^36^ An Italian study performing genotypic characterization of 81 *Klebsiella pneumoniae* carbapenemase (KPC)‐producing *K. pneumoniae* isolates from LT or LuT donors and recipients showed that 83% of them belonged to clonal group 258, in particular to ST258 (20%) and ST512 (60%). The remaining isolates (17%) fell within seven other STs, the most common being ST101.^37^ Another recent study that describes the molecular epidemiology of CP‐KP in 80 recipients from Brazil identified 88.8% and 11.2% of isolates harboring *bla*_KPC−2_ and *bla*_NDM‐1_ respectively. The most common STs were ST11/KPC‐2 (63%), ST437/KPC‐2 (10%) and ST258/KPC‐2 (4%) within clonal complex 11/258 (CC11/258), ST16/KPC‐2 (10%) within CC17, and ST15/NDM‐1 (9%).^38^

The incidence of CRE infection in SOT populations varies considerably across centers and transplant types. Reported rates range from 1% to 16% for LT recipients, 1%–11% for KT recipients, and 1%–8% for LuT recipients.^5^, ^39^ Higher rates of CRE colonization and infection have been reported in studies focused on LT recipients and conducted in centers with established endemicity.^37^, ^39^, ^40^, ^41^ A multinational retrospective cohort (CRECOOLT study) included 840 LT recipients from 15 centers in Italy, Brazil, the USA, Spain, and Israel that had been found to be colonized by CRE either before or at transplantation (24.2%) or within the first 180 days after the procedure (75.8%). The overall infection rate was 29.7%, which occurred at a median of 19 post‐transplant days (range: 9–42).^42^ A prospective monocentric cohort study on 386 LT recipients in Brazil also revealed high incidence rates of CRE colonization (17.6% and 30.8% before or following transplantation), although the proportion of patients that developed infection was lower (15.7% after a median of 11 days).^41^ In a prospective cohort of 553 LT recipients from an Italian center, 7% were pre‐transplant carriers and 19% acquired colonization after the procedure, with a significant increase over the study period (2010–2017).^39^ The overall rate of CRE infection was 10.3%, within a median of 31 days (range: 11–115) from transplantation. This figure rose to 34.7% among patients with previous CRE colonization.^39^ Finally, in a large cohort of KT recipients colonized with CRE, the median time from transplantation to the first CRE‐positive culture was 42 days, and nearly 38% of patients developed an infection, mostly UTI.^43^

Risk factors for CRE infection in LT recipients include CRE carriage acquired before or after transplantation, Model for End‐Stage Liver Disease (MELD) score, multi‐organ transplantation, need for reintervention or RRT, acute kidney injury, prolonged mechanical ventilation, and graft rejection.^39^, ^41^, ^42^, ^44^ Freire et al. found the following risk factors in a single‐center Brazilian cohort of 331 KT recipients with pre‐transplant CRE colonization: recipient age at CRE acquisition >50 years, median lymphocyte count ≤700 cells/mcL, carbapenem use, and colonization by a polymyxin‐resistant strain.^43^ Other risk factors reported in different studies include multi‐organ transplantation, ureteral stent, length of hospital stay, deceased donor allograft, pre‐transplant CR‐KP infection or colonization, diabetes mellitus, and receipt of antimicrobials other than trimethoprim‐sulfamethoxazole.^31^, ^32^

**FIGURE 2:**
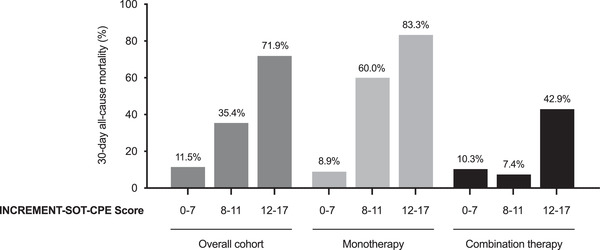
Thirty‐day all‐cause mortality for solid organ transplantation (SOT) recipients with carbapenem‐resistant Enterobacterales and bloodstream infection (CRE BSI) according to increasing categories in the INCREMENT‐SOT‐CPE score and type of active therapy administered (adapted from Pérez‐Nadales et al.^80^)

Optimization of the management of CRE infection relies on reducing the risk of acquisition, improving early detection, and choosing aggressive antibiotic therapy.^45^ In patients with CRE colonization and clinical suspicion of infection, a careful assessment of the underlying conditions and the extent of colonization may serve to guide empirical therapy. As part of the afore‐mentioned CRECOOLT study, Giannella et al. developed a risk prediction model for CRE infection after LT based on the following risk factors: CRE colonization within 60 days before or after transplantation, multisite post‐transplant colonization, prolonged mechanical ventilation, acute kidney injury, and surgical reintervention. The discrimination accuracy of the model in the derivation and bootstrapped validation datasets were acceptable (area under the curve [AUC] of 74.6 and 73.9, respectively).^42^ As part of the INCREMENT‐SOT project, we developed the INCREMENT‐SOT‐CPE score to predict 30‐day all‐cause mortality in SOT recipients with BSI due to CRE.^8^ The score is based on a previously derived and validated tool for the general population^46^, ^47^, ^48^ enriched with additional transplant‐specific variables (i.e., adequate therapy, source control, lymphopenia, and cytomegalovirus [CMV] disease) (Table 1). The score was able to classify patients into three strata according to the mortality risk: 0–7 (low risk), 8–11 (high risk), and 12–17 (very high risk) (Figure 2). Of note, the INCREMENT‐SOT‐CPE score still remains to be externally validated.

**TABLE 1:** The INCREMENT‐SOT‐CPE score for predicting all‐cause 30‐day mortality in solid organ transplantation (SOT) recipients with carbapenem‐resistant Enterobacterales and bloodstream infection (CRE BSI) (adapted from Pérez‐Nadales et al.^80^)

Variable	Regression beta coefficients (95% CI)	Score
INCREMENT‐CPE score ≥8^a^	2.62 (1.79–3.56)	8
CMV disease in the previous 30 days	2.38 (0.58–4.34)	7
Lymphopenia ≤600 cells/mcL	1.24 (0.55–1.97)	4
No source control	0.98 (0.17–1.83)	3
Inappropriate empirical therapy	0.64 (−0.26–1.45)	2
Interaction INCREMENT‐CPE score ≥8 * CMV disease in the previous 30 days^b^	−2.39 (−4.90–−0.10)	−7
Maximum score		17

Abbreviations: CMV, cytomegalovirus; CPE, carbapenemase‐producing Enterobacterales; CRE, carbapenem‐resistant Enterobacterales.

^a^ The INCREMENT‐CPE mortality score was developed to predict all‐cause mortality in carbapenemase‐producing Enterobacterales and includes the following variables: severe sepsis or shock at presentation (5 points), Pitt bacteremia score ≥6 (4 points), Charlson comorbidity index ≥2 (3 points), and source of bloodstream infection other than urinary or biliary tract (3 points).

^b^ The negative interaction coefficient means that the effect of the combined action of 2 predictors is less than the sum of the individual effects. Consequently, in our model, the maximum score in a patient with all risk factors would be 17.

## 4 THERAPEUTIC OPTIONS FOR ESBL‐E AND CRE INFECTIONS IN THE SOT POPULATION

As commented above, the evidence‐based SET/GESITRA‐SEIMC/REIPI document was published in 2018.^3^ This multidisciplinary initiative was justified by the rapid spread across transplant centers of cephalosporin‐ and carbapenem‐resistant strains and by the particular challenges that transplant and infectious diseases physicians face in treating MDR GNB infections. Beyond their increased susceptibility to infection related to long‐term immunosuppression, the SOT population shows distinct clinical characteristics in comparison to non‐transplant patients, such as the repeated exposure to the healthcare system and invasive procedures, the frequent presence of renal or liver impairment associated with nephrotoxic therapies (i.e., calcineurin inhibitors [CNIs]) or underlying conditions, and the risk of drug‐to‐drug interactions. The limited therapeutic options for MDR GNB often require the use of second‐line agents with unacceptable toxicity, such as colistin. On the other hand, the evidence available to guide the management of these difficult‐to‐treat pathogens derives from randomized clinical trials (RCTs) and observational studies that rarely provide separate outcomes for SOT recipients, thus compromising the extrapolation of results.

In this section, we summarize some of the SET/GESITRA‐SEIMC/REIPI recommendations in the light of the new evidence that emerged since their publication, with a focus on the contributions made by the INCREMENT‐SOT consortium for the treatment of ESBL‐E and CRE BSI. The initiatives taken to design carbapenem‐sparing regimens by repositioning existing drugs—either β‐lactams or belonging to other classes—are reviewed. Finally, we summarize the main features and approved indications of the novel antibiotics, as well as the existing experience and their potential positioning in the treatment of post‐transplant infection (Table 2).

**TABLE 2:** Recently approved antibiotic agents with potential activity against extended‐spectrum β‐lactamase‐producing (ESBL‐E) and carbapenem‐resistant Enterobacterales (CRE)

**Agent**	**Approved indications**	**Dose (normal renal function)**	**Experience in SOT recipients**	**Considerations**
Cefiderocol	cUTI, HAP/VAP (FDA) Infections due to aerobic GNB with limited treatment options (EMA)	2 g IV every 8 h	Case reports	The only agent with activity against MBL‐producing CRE FDA warning due to increased all‐cause mortality in phase III RCT compared to best available therapy Potential for resistance selection
Ceftazidime‐avibactam	cUTI, cIAI, HAP/VAP (FDA, EMA) Infections due to aerobic GNB with limited treatment options (EMA)	2/0.5 g IV every 8 h	Case reports, case series, comparative retrospective studies	Activity against OXA‐48‐producing CRE Potential activity against MBL‐producing CRE in combination with aztreonam
Meropenem‐vaborbactam	cUTI (FDA) cUTI, cIAI, HAP/VAP (EMA) Infections due to aerobic GNB with limited treatment options (EMA)	2/2 g IV every 8 h	Case reports	Lower potential for resistance selection than CAZ‐AVI No additional activity beyond meropenem for MDR *Pseudomonas* No activity against OXA‐48‐type and MBL‐producing CRE
Imipenem‐cilastatin‐relebactam	cUTI, cIAI, HAP/VAP (FDA) HAP/VAP, infections due to aerobic GNB with limited treatment options (EMA)	500/500/125 mg IV every 6 h	None	No activity against OXA‐48‐type, GES‐type, and MBL‐producing CRE
Plazomicin	cUTI (FDA)	15 mg/Kg IV every 24 h	None	Potential role of monotherapy Increased risk of nephrotoxicity with calcineurin inhibitors Not available in Europe
Eravacycline	cIAI (FDA, EMA)	1 mg/Kg IV every 24 h	Small case series	More potent activity, better tissue penetration, and lower potential for resistance selection than tigecycline Interaction with strong CYP3A4 inducers Unfavorable PK/PD profile for BSI Should be avoided in cUTI

Abbreviations: BSI, bloodstream infection; CAZ‐AVI, ceftazidime‐avibactam; cIAI, complicated intraabdominal infection; CRE, carbapenem‐resistant Enterobacterales; cUTI, complicated urinary tract infection; EMA, European Medicines Agency; FDA, Food and Drug Administration; HAP/VAP, hospital‐acquired pneumonia/ventilator‐associated pneumonia; MBL, metallo‐β‐lactamase; PK/PD, pharmacokinetic/pharmacodynamic; RCT, randomized clinical trial; SOT, solid organ transplantation.

### 4.1 Old drugs for emerging problems

#### 4.1.1 Repositioning β‐lactams

A notable amount of research has been devoted over the last years to defining the role of non‐carbapenem‐based approaches for ESBL‐E BSI from low‐risk (urinary and biliary) sources, with the aim to spare the use of carbapenems and the subsequent ecological pressure.^49^, ^50^ Existing β‐lactam/β‐lactamase inhibitor combinations (BLBLI) appear as a good alternative since a variable proportion of ESBL‐E isolates across geographical areas (from 20% to over 60%) remain susceptible in vitro to amoxicillin‐clavulanate or piperacillin‐tazobactam.^51^, ^52^, ^53^, ^54^, ^55^, ^56^ Gutiérrez‐Gutiérrez et al. analyzed more than 600 patients with monomicrobial BSI due to ESBL‐E (mainly CTX‐M‐type‐producing *E. coli*) within the INCREMENT project and found a comparable 14‐day cure/improvement rate and 30‐day all‐cause mortality in those receiving active BLBLI or carbapenem monotherapy, either in the empirical or targeted therapy groups. The non‐inferiority of BLBLIs was consistent across different subgroup analyses (e.g., type of ESBL‐E, source of infection, or clinical severity) and after propensity score (PS) adjustment.^57^ These findings were supported by previous, smaller observational studies.^58^, ^59^ Conflictive results, however, had been also reported, with poorer outcomes for BLBLI (particularly with piperacillin‐tazobactam for *K. pneumoniae*).^60^, ^61^, ^62^ Concerns exist on the potential impact of the inoculum effect (i.e., a significant increase in minimal inhibitory concentrations [MICs] with increasing bacterial density), the source of infection, or the coexistence of CTX‐M‐type enzymes with other β‐lactamases (oxacillinase [OXA]‐1/30).^63^ In an attempt to clarify this key question, an open‐label non‐inferiority RCT compared piperacillin‐tazobactam versus meropenem as the definitive therapy for BSI due to ceftriaxone‐resistant *E. coli* or *K. pneumoniae* (MERINO trial). Since the primary outcome of 30‐day all‐cause mortality was more common in the piperacillin‐tazobactam than in the meropenem arm (12.3% vs. 3.7%, respectively), the non‐inferiority assumption could not be demonstrated.^64^ This difference was attenuated, however, when non‐susceptible strains centrally tested by broth microdilution and whole‐genome sequencing were excluded,^65^ or by considering only patients with a urinary tract source or low comorbidity burden.^50^

On the basis of moderate‐quality evidence, the SET/GESITRA‐SEIMC/REIPI document states that carbapenems should be recommended as the empirical or targeted treatment of SOT recipients with moderate‐to‐severe ESBL‐E infections, although BLBLI therapy seems reasonable for nonbacteremic episodes, particularly UTI.^3^ Ongoing immunosuppression and the frequent instrumentation of the urinary tract may limit the extrapolation to the SOT population of results showing the non‐inferiority of BLBLI therapy.^66^ With such uncertainties in mind, we took advantage of the INCREMENT‐SOT Project to compare the effectiveness of active BLBLI‐based monotherapy initiated within the first 72 h from the onset of monomicrobial ESBL‐E BSI secondary to UTI with that of carbapenems in the setting of KT. Sensitivity analyses were conducted at 24‐hour and 7‐day windows. Overall, 306 ESBL‐E BSI episodes were included, with *E. coli* (62.1%) and *Klebsiella* spp. (35.0%) accounting for the majority of isolates. Consistent with the low‐risk nature of the source of infection, therapeutic failure (i.e., lack of cure or clinical improvement and/or death from any cause) was rare (8.2% on day 7 and 13.4% on day 30), as was all‐cause mortality (2.9%). Most patients received carbapenem‐based monotherapy for the first 72 h (68.6%), whereas BLBLI was used in 10.8% of cases only (mostly piperacillin‐tazobactam at 4/0.5 g every 8 h). Hospital‐acquired BSI and the Pitt bacteremia score were independent predictors of therapeutic failure by day 7. We observed no significant differences in the rates of therapeutic failure between KT recipients treated with carbapenem or BLBLI monotherapy, either at day 7 (9.0% vs. 3.0%, respectively; risk difference: ‐6.01%; 95% CI: ‐0.16–0.04; odds ratio [OR]: 3.18; *p*‐value = .267) or day 30 (13.8% vs. 9.1%; risk difference: ‐4.72%; 95% CI: ‐0.17–0.08; OR: 1.60; *p*‐value = .459). Due to baseline imbalances between groups, we constructed a PS for the use of carbapenem monotherapy (that included the geographical area, pre‐transplant heart failure, chronic pulmonary disease, presence of a rapidly or ultimately fatal disease, and early receipt of active therapy). Again, neither the risk of therapeutic failure at day 7 (PS‐adjusted OR: 4.36; 95% CI: 0.51–37.38; *p*‐value = .179) or day 30 (PS‐adjusted OR: 2.59; 95% CI: 0.66–10.21; *p*‐value = .175) were significantly influenced by the type of antibiotic regimen.^11^ +++ Although caution should be exercised in the interpretation of these results due to the low numbers in the BLBLI group and the risk of underpowered sample size, our study would suggest that—provided in vitro susceptibility—BLBLIs may be a reasonable alternative to carbapenems for ESBL‐E BSI from a urinary source in KT recipients, supporting SET/GESITRA‐SEIMC/REIPI recommendations. On the other hand, this conclusion is in line with the results reported for hematological neutropenic patients, including hematopoietic stem cell transplant recipients.^67^ The debate on to what extent carbapenems may be safely spared in favor of BLBLIs is far from being settled,^68^, ^69^ pending on the results of ongoing RCTs.^70^

Another relevant question is whether ertapenem and meropenem are equally effective for ESBL‐E BSI from a urinary source. The increasing prevalence of ESBL producers among uropathogens in KT recipients has contributed to the widespread use of carbapenems.^71^ In contrast to group 2 carbapenems (imipenem, meropenem, or doripenem), ertapenem displays no significant activity against non‐fermenting GNB and would contribute to decrease—or at least not add to—the risk of selective pressure on *Pseudomonas aeruginosa* and *Acinetobacter* baumannii.^72^, ^73^, ^74^ Moreover, ertapenem has more convenient dosing for outpatient parenteral antibiotic therapy (OPAT). Nevertheless, doubts have been raised on the probability of attaining appropriate pharmacokinetic/pharmacodynamic (PK/PD) parameters with the conventional ertapenem dosing in patients with septic shock (due to their increased volume of distribution) or for ESBL‐E isolates different than *E. coli*.^75^ We performed a subanalysis of the INCREMENT‐SOT cohort restricted to KT recipients with bacteremic UTI that received targeted monotherapy with ertapenem or meropenem (100 and 101 patients, respectively). The clinical cure rate by day 14 was comparable between patients treated with ertapenem (45.0%) or meropenem (50.5%), whereas the median hospital stay was shorter in the former group (10.5 vs. 14 days; *p*‐value = .008). In the PS‐adjusted multivariable analysis, targeted therapy with ertapenem was not associated with a lower odds of achieving clinical cure as compared with meropenem (OR: 1.29; 95% CI: 0.51–3.22). This result was confirmed by the inverse probability of treatment weighting and PS‐matching. The desirability of outcome ranking analysis suggested that ertapenem may have some advantages in this setting, such as once‐daily administration, lower ecological impact, and the possibility of OPAT.^9^

#### 4.1.2 The role of combination therapy

**FIGURE 3:**
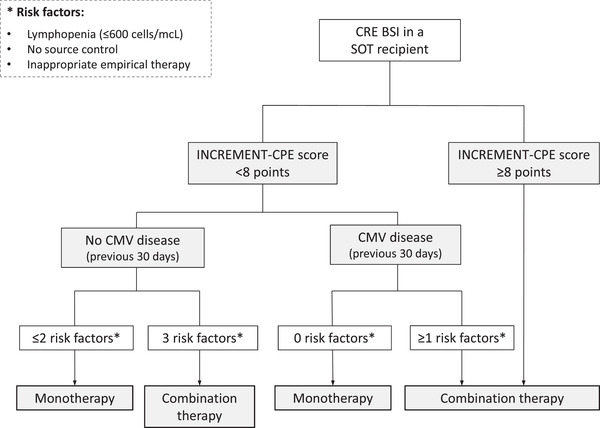
Proposed algorithm for the choice of definitive therapy for post‐transplant CRE BSI (adapted from Pérez‐Nadales et al.^80^). BSI, bloodstream infection; CMV, cytomegalovirus; CRE, carbapenem‐resistant Enterobacterales; SOT, solid organ transplantation

The potential benefit of combination therapy for MDR GNB remains controversial in the non‐transplant population. Available studies are observational in nature and often limited by low sample sizes and heterogeneity in the definition of “combination therapy” (i.e., number of agents, in vitro activity, duration).^7^, ^76^ For instance, a large multicenter study comprising 661 patients with BSI and non‐bacteremic infection due to KPC‐producing *K. pneumoniae* reported that combination therapy with at least two drugs displaying in vitro activity was associated with lower mortality, mainly in patients with septic shock. Meropenem‐containing combinations were effective only if the MIC value of the isolate was ≤8 mg/L.^77^ Some^78^ but not all meta‐analyses^79^ have reported differences in different outcomes—clinical success, microbiological cure, or mortality—between patients treated with monotherapy or combination therapy for CRE. The recent ESCMID guidelines conditionally recommend combination therapy for certain scenarios only, such as severe infections due to metallo‐β‐lactamases (MBL)‐producing CRE or isolates with resistance to new antibiotics.^7^ Existing experience in the SOT setting was much scarce, highlighting the contributions of the INCREMENT‐SOT Consortium. Recipients with CRE BSI treated with a single active agent overall showed increased 30‐day mortality as compared to those receiving combination therapy. This difference, however, was only evident within the highest strata of the INCREMENT‐SOT CPE score (adjusted hazard ratio [aHR] for 12–17 points: 2.82; 95% CI: 1.13–7.06; aHR for 8–11 points: 9.93; 95% CI: 2.08–47.40). In contrast, no apparent benefit derived from the use of combination therapy was observed among patients in the low‐risk stratum (≤7 points) (Figure 2).^80^ These results mirror those obtained from the INCREMENT cohort for non‐transplant patients,^81^ and lead us to propose an algorithm for clinical management (Figure 3). It should be noted that considerable heterogeneity existed in the specific combinations administered, with most of them including an aminoglycoside, colistin, or tigecycline. In addition, the potential impact of “immortal time bias” on the protective effect observed for combination therapy among recipients with higher scores cannot be completely ruled out, although patients dying within the first 48 h after the blood cultures were obtained had been excluded from the analysis.^80^ Finally, these studies were conducted before the introduction of ceftazidime‐avibactam (CAZ‐AVI) or other new BLBLIs reviewed below.

#### 4.1.3 Non β‐lactam alternatives

In addition to BLBLIs, the reprofiling of existing antibiotics has been proposed as an alternative basis for the design of carbapenem‐sparing regimens for ESBL‐E and, in some cases, CRE. These “revisited” drugs include aminoglycosides, tigecycline, fosfomycin, and colistin among others.^50^ All of them are positioned as second‐line regimens by the SET/GESITRA‐SEIMC/REIPI document due to their adverse effects and the increased mortality rate when compared to β‐lactams.^3^

Aminoglycoside monotherapy—with preference given to amikacin—may constitute a valid choice for selected cases with limited therapeutic options, such as non‐bacteremic UTI due to ESBL‐E.^82^, ^83^ In the INCREMENT cohort the use of aminoglycosides as the only active agent for a median of 4 days was not associated with increased mortality in ESBL‐E BSI compared to carbapenems, although the number of patients analyzed was low.^84^ The GESITRA/SEIMC consensus statement on the management of post‐transplant UTI published in 2015 supported monotherapy with gentamicin or amikacin for cystitis due to CRE.^85^ The administration of aminoglycosides for more than 2–3 days among SOT recipients, however, is limited by the development of nephrotoxicity, particularly in the presence of concomitant CNI exposure.^86^

Tigecycline is not affected by β‐lactamases and retains in vitro activity against most ESBL‐E isolates—with the notable exception of the *Morganellaceae* family—and a variable proportion (from 40% to more than 90%) of CRE isolates, including MBLs producers.^87^, ^88^, ^89^, ^90^, ^91^, ^92^ There have been some favorable experiences with tigecycline in the treatment of MDR GNB,^93^, ^94^ and a meta‐analysis pooling data from 21 studies (none of them randomized) suggested efficacy in CRE similar to other antibiotics, particularly as combination therapy or at high doses.^95^ In an Italian study comprising 125 patients with KPC‐producing *K. pneumoniae* BSI, combination therapy with at least two active agents was associated with a lower mortality rate. The best outcomes were obtained with the triple‐drug regimen comprising tigecycline, colistin, and meropenem.^96^ Nevertheless, and likely due to its bacteriostatic action, tigecycline has been consistently shown to be associated with higher mortality when used off‐label to treat serious infections,^97^, ^98^ which resulted in a warning by the Food and Drug Administration (FDA). The low urine concentration achieved with tigecycline prevents its use for UTIs. As for SOT recipients, experience with tigecycline for CRE is mainly limited to single case reports^99^ or small series.^100^, ^101^, ^102^ The SET/GESITRA‐SEIMC/REIPI recommendations state that tigecycline may be considered as the combination drug associated with a carbapenem (provided that the MIC value ≤8 mg/L) for non‐urinary CRE infections, whereas monotherapy should be reserved to non‐severe infections.^3^ After the publication of this document, a single‐center retrospective study found that SOT recipients with polymicrobial IAI treated with tigecycline were less likely to achieve favorable clinical outcomes and experienced more adverse events (AEs) than comparator broad‐spectrum agents.^103^ On the other hand, KT recipients on CNIs may be at an increased risk of developing tigecycline‐induced acute pancreatitis.^104^, ^105^, ^106^

Despite its five decades of existence, fosfomycin has recently regained attention due to its unique mode of action—the irreversible inhibition of the first cytoplasmic step of peptidoglycan synthesis—that results in a potent bactericidal activity with minimal cross‐resistance with other classes of antibiotics.^107^, ^108^ A non‐negligible proportion of ESBL‐E and CRE isolates remain susceptible,^109^ although decreasing rates are being gradually reported.^110^, ^111^, ^112^ Oral formulations (calcium salt and trometamol) have a favorable safety profile, whereas the intravenous fosfomycin disodium is associated with a high sodium intake that may limit its use in patients with heart failure or undergoing hemodialysis.^113^ A multicenter retrospective study performed in Spain included 143 episodes of uncomplicated UTI (cystitis) in KT recipients treated with oral fosfomycin, with rates of clinical and microbiological cure of 83.9% and 70.2%, respectively. Half of the isolates had an MDR phenotype (ESBL‐E in 14.0%, CRE in 3.5%), although the odds of microbiological cure were not decreased in these episodes (71.4%).^114^ Similar results have been also reported for asymptomatic bacteriuria within the first post‐transplant months.^115^ Concordant with these observational experiences, the administration of a single 4‐g dose of intravenous fosfomycin disodium before the placement or removal of a urinary catheter or a double‐J ureteral stent resulted in a lower incidence of symptomatic UTI or asymptomatic bacteriuria during the first weeks after KT as compared with placebo.^116^ Evidence supporting the use of intravenous fosfomycin for serious GNB infection is still emerging.^117^ A recent open‐label RCT compared intravenous fosfomycin versus a β‐lactam (ceftriaxone or meropenem) for patients with BSI from urinary source due to MDR (mainly ESBL‐producing) *E. coli*. While clinical or microbiological failure was lower with fosfomycin than with the comparator (14.3% vs. 19.7%, respectively), the primary outcome of non‐inferiority was not met due to an increased rate of AE‐related discontinuations in the former group, mostly due to the occurrence of heart failure (which happened only in patients over 80 years and those with preexisting heart disease or renal failure).^118^ Although fosfomycin showed high efficacy, this trial exemplifies that patients to be treated with intravenous fosfomycin at doses of 4–6 g every 6–8 h should be properly selected. Moreover, this formulation is not available in some countries. The SET/GESITRA‐SEIMC/REIPI recommendations limit the use of fosfomycin trometamol for non‐severe infections with an adequate site penetration (in particular UTI), whereas intravenous fosfomycin should only be considered for CRE infections as part of a combination regimen which includes at least one more active agent, preferably as three‐drug regimens.^3^

### 4.2 New β‐lactam actors in the MDR landscape

#### 4.2.1 Cefiderocol

Cefiderocol is a third‐generation cephalosporin structurally related to ceftazidime and cefepime, which is conjugated with a catechol moiety on the C‐3 side chain. Similar to other β‐lactams, cefiderocol transits the outer cell membrane of gram‐negative bacteria by passive diffusion through porins. In addition, it actively enters the periplasmic compartment—where high concentrations are achieved—by exploiting the bacterial siderophore‐iron complex pathway as a “Trojan horse”.^119^ This mode of action overcomes common mechanisms of β‐lactam resistance such as loss of porin expression or up‐regulation of efflux pumps and results in stability against hydrolysis by various types of carbapenemases, including most serine enzymes (e.g., KPC, OXA type) and MBLs.^120^ Cefiderocol also retains antibacterial activity against ESBL‐ and AmpC‐producing strains.^121^ Its clinical development program comprised three non‐inferiority phase II/III RCTs.^122^, ^123^, ^124^ One of them compared cefiderocol with the best available therapy (BAT)—as chosen by the investigator and including a maximum of three drugs—in patients with hospital‐acquired pneumonia (HAP), BSI, or cUTI due to carbapenem‐resistant GNB. *Pseudomonas aeruginosa* and *A. baumannii* were the most common pathogens in the microbiological modified intention‐to‐treat (mITT) population. The rate of clinical cure was similar between both groups across the qualifying infectious syndromes. Nevertheless, numerically more deaths occurred in patients receiving cefiderocol (33.7% vs. 18.4% in the BAT arm), mainly in the subgroup with *Acinetobacter* infection.^122^ This unexpected finding led the FDA to issue a warning, although the underlying explanation is unclear and might be related to the heterogeneity in patient characteristics, small sample size, and imbalances between study groups. The remaining two trials demonstrated the non‐inferiority of cefiderocol to high‐dose, extended‐infusion meropenem in terms of all‐cause mortality in patients with HAP,^123^ or to imipenem‐cilastatin in terms of clinical and microbiological outcomes in cUTI.^124^ The safety profile observed is concordant with that expected for cephalosporins, and dose adjustment is required in moderate to severe renal impairment.^119^ Cefiderocol was FDA approved in 2019 for the treatment of cUTI (including pyelonephritis) and in 2020 for HAP and ventilator‐associated pneumonia (VAP). The European Medicines Agency (EMA) granted a generic approval for infections due to aerobic GNBs with limited therapeutic options. A common feature of the novel agents for the treatment of ESBL‐E and CRE reviewed herein is the scarcity of data available for the specific population of SOT recipients. This limitation also applies for cefiderocol, with only a few cases reported.^125^, ^126^, ^127^, ^128^ A LT recipient that developed liver abscesses and BSI due to KPC‐producing *K. pneumoniae* resistant to CAZ‐AVI was successfully treated with cefiderocol for >10 days.^126^ AKT recipient was treated with cefiderocol in combination with CAZ‐AVI a polymyxin B for a complicated IAI (cIAI) due to two genetically different *K. pneumoniae* strains carrying *bla*_NDM‐1_, *bla*_OXA‐232,_ and *bla*_CTX‐M‐15_ genes, with bacterial clearance.^125^ It has been also reported the case of an LT recipient with cUTI due to an MDR *C. freundii* strain harboring *bla*_KPC‐1_ and *bla*_NDM‐3_ that was extensively resistant to CAZ‐AVI (MIC >256 mg/L), in which clinical cure was achieved after a 10‐day course of cefiderocol (MIC = 1 mg/L).^127^ The rapid development of high‐level resistance in *E. cloacae* within the first weeks of cefiderocol therapy has been recently reported in an LT recipient. Whole‐genome sequencing identified functional alterations in the *cirA* gene encoding for the catecholate siderophore receptor used by the cefiderocol‐ferric complex to enter the periplasmic compartment.^128^ Pending the generation of clinical experience, cefiderocol appears as a promising option for MDR GNB infection in SOT recipients.

#### 4.2.2 New BLBLI combinations

Three new BLBLIs have been recently added to the therapeutic armamentarium against MDR GNB: CAZ‐AVI, meropenem‐vaborbactam (MER‐VAB), and imipenem‐cilastatin‐relebactam (IMI‐REL).^129^, ^130^, ^131^ All of them retain activity against ESBL‐E and AmpC‐producing Enterobacterales, although some important differences must be noted. Avibactam is the first member of the diazabicyclooctanone non‐β‐lactam class and exhibits the broadest spectrum of β‐lactamase inhibition, including Ambler class A (e.g., KPC‐ and Guiana extended‐spectrum [GES]‐type), class C (Amp‐C cephalosporinases), and some class D enzymes (notably OXA‐48‐like).^132^ The FDA approved CAZ‐AVI in 2015 for the treatment of cUTI and cIAI, and in 2018 for patients with HAP/VAP. Soon after its release, descriptions emerged of the development of CAZ‐AVI resistance in KPC‐producing strains, typically driven by the D179Y substitution in the *bla*_KPC‐3_ gene that affects the stability of the Ω‐loop.^133^, ^134^ Vaborbactam is a new cyclic boronate β‐lactamase inhibitor with potent activity against KPC‐2 and KPC‐3 enzymes, as well as ESBLs (e.g., sulfhydryl variable, temonera, CTX‐M)^135^ and class C cephalosporinases.^130^ Vaborbactam is also able to overcome CAZ‐AVI resistance due to D179Y mutations at the KPC binding site.^136^ Of note, carbapenem‐hydrolyzing class D enzymes are not affected by vaborbactam,^137^ a notable limitation in European countries such as Spain where OXA‐48‐like enzymes are the most common type of carbapenemase.^138^, ^139^ Resistance to MER‐VAB among KPC‐producing isolates is mediated by a combination of KPC production and mutations in the OmpK35 and OmpK36 porins, although the odds of resistance developing seem to be lower than with CAZ‐AVI.^140^, ^141^ Finally, relebactam is also a diazabicyclooctane β‐lactamase inhibitor, like avibactam, that bears a piperidine ring in the R1 side chain.^131^, ^142^ Similar to MER‐VAB, IMI‐REL is highly effective against CRE isolates carrying *bla*_KPC‐2_ and *bla*_KPC‐3_ genes and ESBL‐E and AmpC‐producing Enterobacterales. Nevertheless, the inhibitory activity against most OXA‐type and GES‐type carbapenemase‐producing strains is poor.^143^, ^144^ It should be highlighted that none of these novel BLBLI combinations retain significant activity in the presence of MBLs.^129^

Experience with new BLBLIs among SOT recipients has been mainly restricted to case reports^145^, ^146^, ^147^, ^148^ and small series with no comparator group.^149^, ^150^ The SET/GESITRA‐SEIMC/REIPI document did not make any mention of MER‐VAB or IMI‐REL and simply suggested—based on low‐quality evidence (CIII)—that CAZ‐AVI may be considered for the treatment of CRE infections if the strain shows in vitro susceptibility.^3^ In view of this research gap, we have conducted a subanalysis within the INCREMENT‐SOT cohort in which the effectiveness of CAZ‐AVI was compared with BAT (mainly including colistin, tigecycline, or fosfomycin) in 210 recipients diagnosed with monomicrobial CRE BSI from 2016 to 2021. In the primarily targeted therapy cohort, 85 and 81 patients were analyzed within the CAZ‐AVI and BAT groups, respectively. There were significant differences favoring CAZ‐AVI over BAT in the 14‐day (81.2% vs. 58.0%, *p* = .001) and 30‐day (81.2% vs. 60.5%, *p* = .004) clinical success rate as well as in 30‐day mortality (12.9% vs. 27.2%, *P* = 0.036) that persisted after multivariate adjustment. Interestingly, we were able to validate in this cohort the good performance of the INCREMENT‐SOT CPE Score to predict the risk of death (adjusted OR per one‐point increment: 1.17; 95% CI: 1.08–1.28, *p* < 0.001) (data not yet published).

### 4.3 Non‐β‐lactam classes or the promising newcomers

#### 4.3.1 Plazomicin

Plazomicin is a novel semisynthetic parenteral aminoglycoside that inhibits bacterial protein synthesis.^151^ Thanks to various structural modifications it retains activity in the presence of most aminoglycoside modifying enzymes, as well as ESBLs and carbapenemases (including MBLs).^151^, ^152^, ^153^ A phase III RCT showed the non‐inferiority of plazomicin to meropenem for cUTI. The rate of microbiological eradication at the test‐of‐cure visit in patients with ESBL‐E infection was numerically higher in the plazomicin group (82.4% vs. 75.0%, respectively).^154^ A second small open‐label trial recruited patients with CRE BSI or HAP/VAP that were randomized to receive plazomicin or colistin in combination with meropenem or tigecycline. The composite outcome of death from any cause at 28 days or disease‐related complications in the microbiological mITT population was numerically lower with plazomicin than colistin. Such a benefit was observed only in the subgroup of patients with BSI, although the numbers were too low to draw reliable conclusions since the trial was prematurely terminated due to slow enrollment.^155^ Plazomicin received FDA approval in 2018 for adult patients with cUTI, whereas the application for marketing authorization in Europe was withdrawn by the manufacturer in June 2020. Unfortunately, clinical experience in SOT recipients is still lacking. Of note, this population was excluded from the pivotal trial for cUTI as per study protocol.^154^ Similar to other aminoglycosides, the risk of nephrotoxicity remains a concern with plazomicin, in particular among patients with impaired baseline renal function and higher cumulative drug exposure.^154^ This safety profile would further limit its use in the KT setting. It is likely that therapeutic drug monitoring will be needed to optimize exposure and clinical outcomes.^156^ Other AEs observed include ototoxicity, gastrointestinal disturbances, and hypotension.^151^

#### 4.3.2 Eravacycline

Eravacycline is a novel, fully synthetic fluorocycline that reversibly binds to the 30S ribosomal subunit, blocking the elongation phase of bacterial protein synthesis. In line with other members of the tetracycline class, eravacycline shows a broad spectrum of in vitro activity that comprises methicillin‐resistant *Staphylococcus aureus*, vancomycin‐resistant enterococci, anaerobes, *A. baumannii* and some ESBL‐E and CRE.^157^ Susceptibility rates among CRE isolates range from 96.2% to 98.0% for *E. coli* and from 66.6% to 84.0% for *K. pneumoniae*.^158^, ^159^, ^160^, ^161^ Interestingly, eravacycline shows reasonable activity against New Delhi MBL (NDM)‐producing Enterobacterales, with a reported susceptibility rate of 66.2% according to the CLSI interpretive criteria,^162^ and has been shown to be 2‐ to 4‐fold more active than tigecycline against CRE.^163^ In addition, it remains active against the main acquired tetracycline‐specific resistance mechanisms (efflux pumps and ribosomal protection proteins).^163^ On the basis of two non‐inferiority phase III RCTs,^164^, ^165^ intravenous eravacycline was approved by the FDA and EMA in 2018 for the treatment of cIAI. Eravacycline requires dose adjustment in severe liver function impairment (Child‐Pugh class C) and with the concomitant use of strong CYP3A4 inducers. In addition to its improved activity, eravacycline offers advantages over tigecycline, such as higher serum and tissue concentrations, the existence of both oral and intravenous formulations, and better gastrointestinal tolerability.^157^ Again, the available experience in SOT is very limited, as this condition was considered an exclusion criterion in pivotal trials.^164^, ^165^ A retrospective study with 66 patients from three US centers that received eravacycline for a mean of 13.1 days—mainly as monotherapy for off‐label indications such as pneumonia or skin and soft tissue infection—included 7 SOT recipients (10.6%). The overall rate of clinical improvement was 95.5%, although outcomes for the SOT group were not separately provided.^166^ The high volume of distribution of eravacycline (58.3–320.0 L) confers a theoretically unfavorable PK/PD profile for treating BSI, although the pooled clinical and microbiologic outcomes in patients with bacteremic cIAI recruited in the pivotal trials were similar to those observed in the carbapenem arms.^167^

## 5 CONCLUSIONS AND FUTURE RESEARCH AVENUES

As outlined in the present review, MDR GNB constitutes a rapidly evolving threat to modern medicine that adopts particular epidemiological, clinical, and therapeutic features in the SOT population. Notable advances have been made in the management of ESBL‐E and CRE infections after SOT since the publication, 4 years ago, of the SET/GESITRA‐SEIMC/REIPI recommendations.^3^ The findings generated by the INCREMENT‐SOT consortium have helped to refine the prognostic stratification in SOT recipients with CRE BSI—highlighting the impact of previous CMV disease and lymphopenia—as well as the role of antibiotic combination therapy in this setting.^80^ Other relevant contributions include the comparable effectiveness of active BLBLI‐based monotherapy versus carbapenems for ESBL‐E BSI in KT recipients,^11^+++ and the potential advantages of ertapenem compared to group 2 carbapenems to minimize selective antibiotic pressure in such patients^9^. Beyond the reprofiling of existing antibiotics (such as aminoglycosides, tigecycline, or fosfomycin) to spare the use of carbapenems against ESBL‐E or to provide alternative agents in CRE infection, recent years have witnessed the approval of novel β‐lactam and non‐β‐lactam agents. In view of their activity in the presence of most serine carbapenemases and ESBLs and their favorable PK/PD and safety profiles, newer BLBLIs combinations and cefiderocol are particularly promising. The clinical experience reported with these agents in SOT recipients (summarized in Table 3), however, is so far limited. In this line, the INCREMENT‐SOT Project is generating real‐life data for CAZ‐AVI in the treatment of post‐transplant CRE BSI. Despite these achievements, many challenges persist. In particular, the development of effective agents that retain activity against MBL‐producing Enterobacterales represents an unmet need. It must be highlighted that the literature supporting the clinical benefit of combination therapy for patients with more severe infections—higher INCREMENT‐SOT‐CPE scores—was produced before the novel therapeutic options became available (particularly new BLBLIs). Therefore, further studies should be conducted to eventually confirm such findings. A better understanding is required of how immunosuppression should be managed in recipients with serious ESBL‐E and CRE infections (e.g., BSI, cIAI, or HAP/VAP) and the long‐term consequences on graft function. In addition, robust evidence derived from RCTs to guide the treatment of ESBL‐E and CRE infections after SOT is still essentially lacking. The INCREMENT‐SOT Cohort exemplifies the usefulness of multinational initiatives specifically aimed at providing high‐quality observational data on the optimal therapeutic approach to MDR GNB infections in the SOT population.

**TABLE 3:** Summary of the reported experience with novel antibiotic agents for the treatment of solid organ transplantation (SOT) recipients with extended‐spectrum β‐lactamase‐producing (ESBL‐E) and carbapenem‐resistant Enterobacterales (CRE) infections

**Agent, reference**	**Type of SOT**	**Type of infection**	**Isolate, molecular resistance mechanism (if available)**	**Outcomes**
Cefiderocol^125^	KT recipient	BSI and cIAI following allograft nephrectomy	Two different carbapenem‐resistant *Klebsiella pneumoniae* strains (ST14 and ST2497) carrying *bla*_NDM‐1_, *bla*_OXA‐232_, *bla*_CTX‐M‐15_, *armA*, and *tet(D)*	Clinical and microbiological response Non‐related death (ischemic colitis and multiorgan failure)
Cefiderocol^126^	LT recipient	BSI and cIAI (liver abscesses)	Carbapenem‐resistant *K. pneumoniae*	Clinical and microbiological response
Cefiderocol^127^	LT recipient	cUTI	*Citrobacter freundii* complex carrying *bla*_NDM‐1_ and *bla*_KPC‐3_	Clinical and microbiological response Non‐related death (angiosarcoma)
Cefiderocol^128^	LT recipient	BSI and cIAI (multiple biloma and hepatic abscesses)	Carbapenem‐resistant *Enterobacter cloacae* (ST96) carrying *bla*_NDM‐5_ and *bla*_OXA‐48_	Initial microbiological response, the emergence of resistant isolate (mutations in *cirA* gene) leading to relapse of infection and death
Meropenem‐vaborbactam^145^	LT recipient	BSI and cIAI (liver abscess due to hepatic artery thrombosis)	Carbapenem‐ and CAZ‐AVI‐resistant *K. pneumoniae* carrying mutant *bla*_KPC‐2_ with D179Y mutation within the KPC Ω‐loop	Clinical and microbiological response
Ceftazidime‐avibactam^146^	KT recipient	Recurrent UTI	Carbapenem‐resistant *K. pneumoniae*	Clinical and microbiological response
Ceftazidime‐avibactam^147^	KT recipient	BSI, cUTI, and pneumonia (probable donor‐derived infection)	Carbapenem‐resistant *K. pneumoniae* carrying *bla*_KPC‐2_	Initial clinical and microbiological response, relapse of BSI after 11 days with an appropriate response after a second course
Ceftazidime‐avibactam^148^	KT recipient	Vertebral osteomyelitis	Carbapenem‐resistant *K. pneumoniae* carrying *bla*_KPC_	Clinical response
Ceftazidime‐avibactam^149^	LuT recipients (*n* = 10)	Pneumonia and/or tracheobronchitis (*n* = 9), BSI and cIAI (*n* = 1)	Carbapenem‐resistant K. pneumoniae carrying *bla*_KPC‐2_	Clinical and microbiological response in 9/10 (90%) Treatment failure in 1/10 (10%) Relapse of lower respiratory tract infection in 5/10 (50%) 30‐ and 90‐day survival of 100% and 90%
Ceftazidime‐avibactam^150^	LuT recipients (*n* = 4)	Pneumonia (*n* = 10)	MDR *K. pneumoniae*	Clinical and microbiological response in 10/10 (100%)
Eravacycline^166^	SOT recipients (*n* = 7) within a larger cohort (*n* = 66)	NA for SOT recipients	MDR GNB or GPC (NA for SOT recipients)	NA for SOT recipients *Overall cohort*: Clinical response in 63/66 (95.5%) Treatment‐emergent adverse events in 3/66 (4.5%)

Abbreviations: BSI, bloodstream infection; CAZ‐AVI, ceftazidime‐avibactam; cIAI, complicated intraabdominal infection; cUTI, complicated urinary tract infection; GNB, gram‐negative bacilli; GPC, gram‐positive cocci; KT, kidney transplantation; LT, liver transplantation; LuT, lung transplantation; MDR, multidrug‐resistant; NA, not available; SOT, solid organ transplantation.

## CONFLICT OF INTEREST

The authors declare no conflict of interest.

## AUTHOR CONTRIBUTIONS

Elena Pérez‐Nadales, Mario Fernández‐Ruiz, and Julian Torre‐Cisneros conceived the review study. Elena Pérez‐Nadales and Mario Fernández‐Ruiz drafted the manuscript. Belén Gutiérrez‐Gutiérrez, Álvaro Pascual, Jesús Rodríguez‐Baño, Luis Martínez‐Martínez, José María Aguado, and Julian Torre‐Cisneros critically reviewed the manuscript.
